# Treatment of Bursal Disease of the Knee-joint

**Published:** 1847-01

**Authors:** John Richard

**Affiliations:** Leamington


*Treatment of Bursal Disease of the Knee-joint.* By JOHN RICHARD, Esq., of Leamington.—The plan of passing a bit of sewing-silk through the centre of the tumour I have practised for the last fifteen years. It has never failed in my hands, and I do not think that I have ever allowed the silk to remain longer than through the third day. When the inflammation has been very considerable, I have always enjoined rest, and applied a few leeches, followed up by a liq. plumbi lotion, previous to introducing the silk.—*Ibid.*

